# Metalorganic chemical vapor deposition growth of InAs/GaSb type II superlattices with controllable As*_x_*Sb_1-*x *_interfaces

**DOI:** 10.1186/1556-276X-7-160

**Published:** 2012-02-28

**Authors:** Li-Gong Li, Shu-Man Liu, Shuai Luo, Tao Yang, Li-Jun Wang, Feng-Qi Liu, Xiao-Ling Ye, Bo Xu, Zhan-Guo Wang

**Affiliations:** 1 Key Laboratory of Semiconductor Materials Science, Institute of Semiconductors, Chinese Academy of Sciences, Beijing, 100083, People's Republic of China; 2 Department of Physics, Tsinghua University, Beijing, 100084, People's Republic of China

## Abstract

InAs/GaSb type II superlattices were grown on (100) GaSb substrates by metalorganic chemical vapor deposition (MOCVD). A plane of mixed As and Sb atoms connecting the InAs and GaSb layers was introduced to compensate the tensile strain created by the InAs layer in the SL. Characterizations of the samples by atomic force microscopy and high-resolution X-ray diffraction demonstrate flat surface morphology and good crystalline quality. The lattice mismatch of approximately 0.18% between the SL and GaSb substrate is small compared to the MOCVD-grown supperlattice samples reported to date in the literature. Considerable optical absorption in 2- to 8-μm infrared region has been realized.

**PACS: **78.67.Pt; 81.15.Gh; 63.22.Np; 81.05.Ea

## Introduction

InAs/GaSb superlattices (SLs) are important for long-wavelength infrared (IR) applications because of their broken gap type II band alignment with the conduction band minimum of InAs lying below the valence band maximum of GaSb. Such type II SL material has been investigated widely as a viable alternative to conventional HgCdTe IR detecting materials due to the unique capability for band structure engineering which results in great flexibility in controlling the detection wavelength (from 3 to 30 μm) [1], low Auger recombination rates [2,3] and small tunneling current [4]. Recently, quantum well IR photodetectors (QWIPs) [5-7] have also exhibited a number of potential advantages including highly uniform and well-controlled epitaxy growth, but their quatum efficiency cannot compete with HgCdTe photodiode due to the fact that optical transition is forbidden for normal incidence of light. More recently, new nanostructured IR photodectors based on quantum dots have been investigated intensively to outperform QWIPs since they are intrinsic sensitive to normal incidence light [8-10]. However, the absorption efficiency is still low due to the limited density of dots and inhomogeneous dot size. In the case of InAs/GaSb SL structures, the absorption is strong for normal incidence of light. Consequently, the SL structures provide the possibility to have both high absorption efficiency as reached with HgCdTe and high uniform as reached with QWIPs. So far, high-quality InAs/GaSb materials have been grown by molecular beam epitaxy (MBE) [11-13]. However, the capability to grow device-quality material and structure by metalorganic chemical vapor deposition (MOCVD) has not been fully achieved, which is the preferred growth technique in manufacturing due to the higher growth rate and multi-wafer capability. Reports on the MOCVD growth of InAs/GaSb are scarce in the literature [14,15].

One of the key issues during the growth of InAs/GaSb SLs is to introduce suitable interfacial layers between InAs and GaSb to offset their lattice mismatch of 0.59%, since misfit dislocations will be generated as the number of layers in the SL increases. The widely investigated MBE growth of such SLs usually introduces InSb-like interface (IF) to make the SL match the GaSb substrate as much as possible [11,12,16]. However, we have shown that good-quality InSb cannot be obtained since InSb is unstable at the optimal temperature for the MOCVD growth of InAs/GaSb SLs [17]. Even though GaAs-like IFs should be stable during MOCVD growth, SLs with GaAs-like IFs have poor crystalline quality since GaAs has a lattice 8% smaller than GaSb substrate, leading to a large lattice mismatch between the SL and GaSb substrate [18,19]. Therefore, new types of IFs more stable than InSb with larger lattice constants than GaAs are necessary for improvement of MOCVD grown InAs/GaSb SL materials. In our recent work, ternary alloy IFs with mixed As and Sb were introduced in our MOCVD growth and have been demonstrated to improve material quality, significantly compared to InSb-like and GaAs-like IFs. Here, we report the growth and characterization of InAs/GaSb SLs on (100) GaSb substrates with an As*_x_*Sb_1-*x *_mixing plane that connects the InAs and GaSb layers as the interfacial layers.

## Experimental details

InAs/GaSb SLs were grown on (100) GaSb substrates in an Aixtron (AIXTRON Ltd. Nanoinstruments, Swavesey, Cambridge, UK) MOCVD reactor system equipped with a close-coupled showerhead growth chamber and an EpiTT optical *in situ *sensor. The chamber pressure during growth was 100 Torr. Epi-pure™ (Epichem Inc., Haverhill, MA, USA) trimethylindium and triethylgallium were used as column III precursors and trimethylantimony (TMSb) and arsine (AsH_3_) were used as column V precursors. Prior to the growth, the substrates were cleaned in HCl to remove native surface oxide and then rinsed in isopropyl alcohol followed by N_2 _blow-drying. In a typical growth, 100-nm GaSb buffer was firstly deposited at 580°C, then the temperature was ramped down to 520°C for the growth of InAs/GaSb SL. Here, we grew 100-period SLs with nominal structures of 4.5-nm InAs and 3-nm GaSb with a plane of mixed As and Sb atoms connecting the GaSb and InAs as the IF layer. The growth of the IFs was controlled by atomic layer epitaxy-like switching sequence [20]. The nominal *x *value of As*_x_*Sb_1-*x *_is 0.18. To obtain the AsSb mixing planes, a 3-s growth interruption with AsH_3 _flowing was introduced after the growth of InAs layer, then the AsH_3 _and TMSb switching valves were simultaneously closed and opened, respectively. The TMSb switching valve was left open for 10 s to form the nominal As_0.18_Sb_0.82 _plane.

The growth was resumed with the deposition of GaSb. The IF, thus, formed is a GaSb-on-InAs IF. For an InAs-on-GaSb IF, the GaSb surface was firstly smoothed by TMSb flowing for 0.5 s, then As was introduced for 5 s to exchange desired amount of Sb atoms on the GaSb surface followed by InAs deposition. The growth rate was 0.7 Å/s for InAs and 1 Å/s for GaSb layers, respectively. The surface morphologies of InAs/GaSb SLs were characterized by atomic force microscopy (AFM, Solver P47) operating in air at room temperature. High-resolution X-ray diffraction (HRXRD, Bede D1) was carried out with Cu Kα_1 _radiation as the source. Polarized Raman scattering measurements were performed in the backscattering geometry at room temperature with a Jobin Yvon (HORIBA Jobin Yvon Inc., Edison, NJ, USA) HR800 confocal micro-Raman spectrometer. Three scattering configurations, $X\left(Y^{\prime },Y^{\prime }\right)\bar{X}$, $X\left(Z^{\prime },Z^{\prime }\right)\bar{X}$ and $X\left(Y^{\prime },Z^{\prime }\right)\bar{X}$, were employed to assist in identifying the nature of the IF modes. *X*, *Y*' and *Z*' are defined along the $\left[\bar{1}00\right]$, [011] and $\left[01\bar{1}\right]$ crystallographic directions, respectively. The sample was excited by the 514.5-nm line of an Ar-ion laser to a 1-μm spot on the surface. IR transmission spectra were measured at room temperature using an IFS120HR Fourier-transform infrared (FTIR) spectrometer.

## Results and discussion

**Figure 1:**
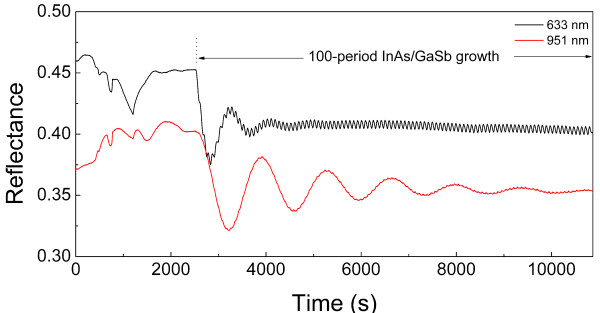
**Example of *in situ *reflectance oscillations versus time**. *In situ *reflectance oscillations versus time at 633 (upper black line) and 951 nm (lower red line) obtained for InAs/GaSb SL growth on GaSb substrate.

*In situ *reflectance can give us useful information of the growth surface during the epitaxy. Figure 1 shows the *in situ *reflectance at 633 and 951 nm of a 100-period InAs/GaSb SL sample. Fabry-Perot (FP) oscillations are visible in both reflectance curves during the SL growth. The oscillation amplitude of the 951-nm reflectance damps gradually with the growth time due to the gradually increased absorption of the SL material. Using a VI mode [21], the period of the SL structure extracted from this reflectance curve is 7.5 nm. For the 633-nm reflectance signal, the FP oscillation damps very quickly since the absorption of the SL material is significant at 633 nm. However, a high frequency and uniform oscillation can be clearly observed on the 633-nm reflectance curve, which arises from the different reflectance of InAs and GaSb. It is known that the intensity of the mirror-reflectance signal is related to the morphology of the growth surface; thus, the flat reflectance curve with the reflectance above 0.4 indicates rather smooth growth surface during the epitaxy of the SL structure.

**Figure 2:**
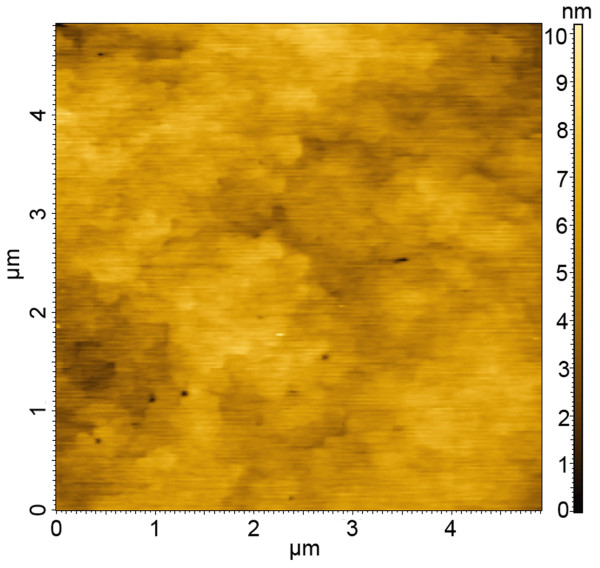
**AFM image of a 100-period InAs/GaSb SL sample for 5 × 5 μm^2 ^scan area**.

The smooth surface of the sample is also confirmed by the AFM image shown in Figure 2. The root mean square (RMS) surface roughness value of the sample was about 0.7 nm over 5 × 5 μm^2 ^scan area. Misfit dislocations due to strain accumulation with the thickness of the grown layer did not generate in our 100-period SL samples. Therefore, according to the *in situ *monitoring reflectance and the AFM image, the planes of mixed As and Sb atoms that we introduced between InAs and GaSb have offset at least partly the strain arising from the lattice mismatch of 0.6% between InAs and GaSb as we expected.

**Figure 3:**
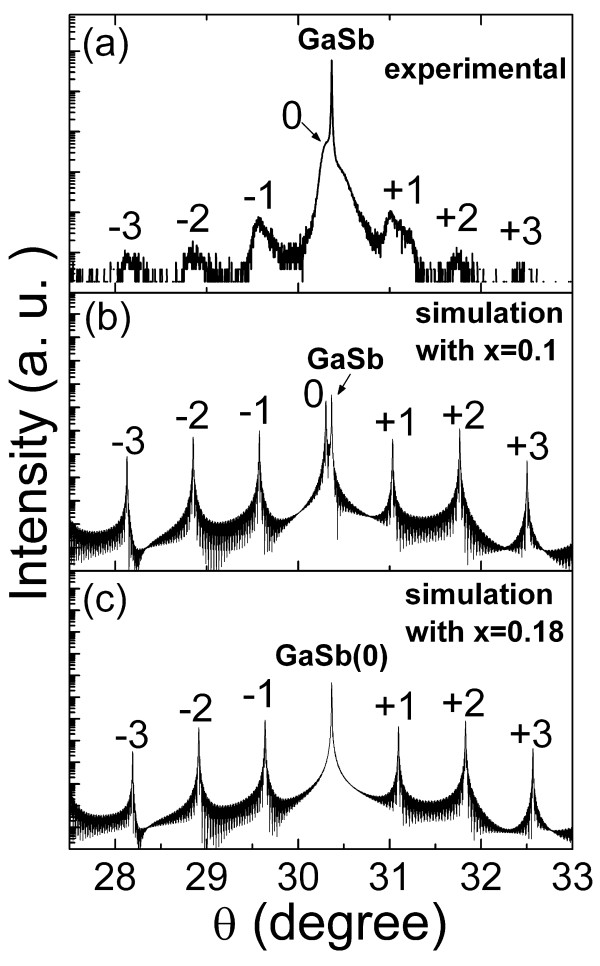
**Crystalline properties of the samples characterized by HRXRD**. Experimental XRD pattern of a 100-period InAs/GaSb SL sample (a). Dynamical simulation to pattern (a) with the fitting parameter *x *= 0.1 (b). Dynamical simulation with the fitting parameter *x *= 0.18, making the SL match the completely GaSb substrate (c).

The crystalline properties of the samples were characterized by HRXRD, and a typical pattern around the (004) reflection of GaSb substrate is shown in Figure 3 (a). Several well-resolved SL satellite peaks are observed in addition to the GaSb substrate, indicating high crystalline quality of the SL structure. The period as measured by the fringes spacing of the satellite peaks is 7.5 nm, in good agreement with the result of the in-situ reflectance. It is known that the position of the zeroth-order satellite peak is related to the average lattice constant of the SL via Bragg's law. The angle separation of peaks between the SL zeroth-order and GaSb substrate is only approximately 216 arc sec, corresponding to the mismatch of only approximately 0.18% between the average lattice constant of the SL and the GaSb substrate. This value is comparable to the best result reported to date in the literature on the MOCVD growth of InAs/GaSb SL [22]. We also conducted the dynamical simulation to the XRD data using the *x *value of As*_x_*Sb_1-*x *_as the fitting parameter. Figure 3 (b) and 3(c) shows two simulated curves for *x *= 0.1 and *x *= 0.18, respectively. It is seen that the zeroth satellite peak overlaps completely with the GaSb (004) peak, i.e., achieving complete strain compensation when *x *is set to 0.18. Here, the experiment XRD pattern can be fitted well by *x *= 0.1, close to the optimal 0.18, indicating nearly complete strain compensation in our SL samples.

**Figure 4:**
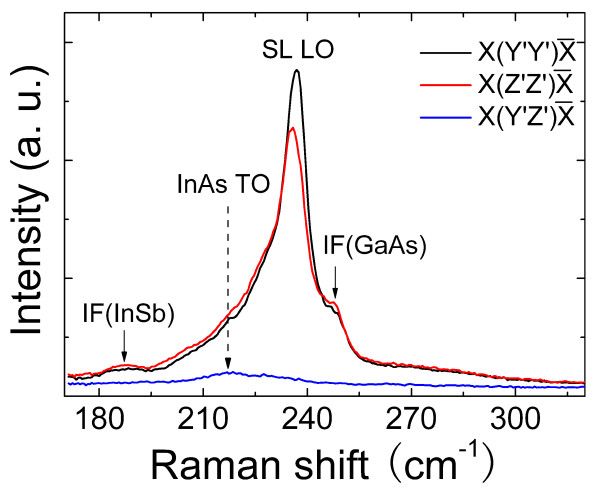
**Room-temperature Raman scattering spectra of a 100-period InAs/GaSb SL sample**. Spectra are shown for the $X\left(Y^{\prime },Y^{\prime }\right)\bar{X}$, $X\left(Z^{\prime },Z^{\prime }\right)\bar{X}$ and $X\left(Y^{\prime },Z^{\prime }\right)\bar{X}$ configurations.

The interfaces in InAs/GaSb superlattices are different from that in usual III-V SLs such as GaAs/AlGaAs. The change of both group III and group V atoms across the IFs leads to the appearance of GaAs-like or InSb-like bonds at the interface, which do not present in both InAs and GaSb [23,24]. Because the vibrational frequencies of these bonds occur in energy ranges that do not support propagating modes in either the GaSb or InAs layers, IF vibrational modes localized at the interfaces are formed. The vibrational energy and intensity of these localized IF modes, especially the GaAs-like IF mode, are very sensitive to the local environments; therefore, Raman scattering by IF modes provides a direct assessment of the IF bonds formed [25-27]. Figure 4 shows the room-temperature Raman scattering spectra of one sample measured in three configurations. The strongest feature measured in the $X\left(Y^{\prime },Y^{\prime }\right)\bar{X}$ and $X\left(Z^{\prime },Z^{\prime }\right)\bar{X}$ configurations is at near 236 cm^-1^, arising from the quasi-confined longitudinal-optical (LO) phonon modes of GaSb and InAs. Because of the overlap in the frequencies of the LO modes of GaSb and InAs, we are unable to distinguish the LO modes of these two materials. In addition, there are two weak peaks in both $X\left(Y^{\prime },Y^{\prime }\right)\bar{X}$ and $X\left(Z^{\prime },Z^{\prime }\right)\bar{X}$ configurations at 248 and 188 cm^-1^. The 248-cm^-1 ^peak is assigned to the strongly localized Ga-As longitudinal IF vibration, and the very weak 188-cm^-1 ^peak is assigned to the weakly localized In-Sb longitudinal IF vibration. According to the calculation with a random-element isodisplacement model by Shanabrook et al. [28], the vibrational energy of 248 cm^-1 ^in Figure 4 could be attributed to an As_0.6_Sb_0.4 _plane. This result is different from our dynamical simulation where the As composition is 0.1. This difference is reasonable due to the inherent simplicity of the model as mentioned by the authors. In the $X\left(Y^{\prime },Z^{\prime }\right)\bar{X}$ configuration, all the Raman vibration modes are forbidden in the backscattering geometry from the (100) surface. However, a weak peak at 218 cm^-1 ^is still visible which is assigned to the transverse optical vibrations confined in the InAs [29]. The activation of this forbidden mode could result from some strain-related effects.

**Figure 5:**
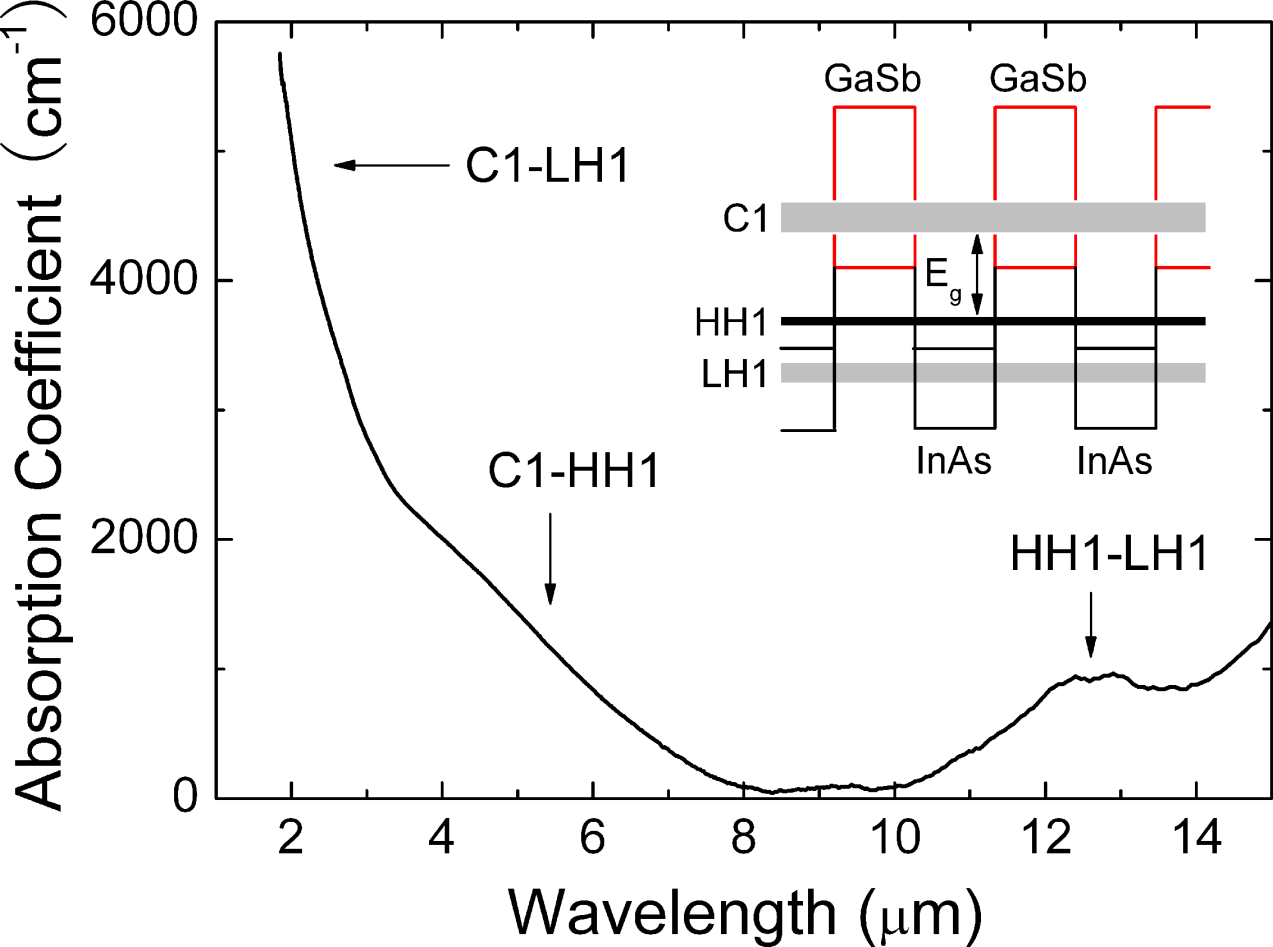
**Absorption spectrum of a 100-period InAs/GaSb SL sample**. The inset is the schematic band edge alignment of constituent layers (InAs and GaSb) and SL sub-bands in InAs/GaSb SL.

Our 100-period SL samples show considerable IR absorption in the wavelength range of 2 to8 μm as shown in Figure 5. The absorption spectrum was extracted from the FTIR transmission spectra measured at room temperature of an InAs/GaSb SL sample. Substrate absorption has been eliminated from the experimental data by subtracting substrate spectrum derived from prior spectrum test of the substrate itself. The absorption coefficient in this range is comparable to those of high-quality SLs of this thickness grown by MBE [30]. The absorption spectrum exhibits a sharp band edge and a series of features associated with the transitions between first electron (C1) and first heavy hole (HH1) sub-bands, C1 and first light hole (LH1) sub-bands, and HH1 and LH1 sub-bands as shown in the inset of Figure 5.

## Conclusion

We have achieved 100-period InAs/GaSb SLs on (100) GaSb substrates by MOCVD. An As_0.1_Sb_0.9 _plane connecting InAs and GaSb was introduced as the IF layer to compensate the strain of the SL to the GaSb substrate. The validity of the strain compensation was confirmed by the results of the *in situ *reflectance, AFM, HRXRD and Raman scattering measurements. The RMS roughness of the surface is 0.7 nm, and the lattice mismatch between the SL and the substrate is 0.18%. Absorption coefficient of approximately 2,000 cm^-1 ^is realized in mid-IR region.

## Abbreviations

AFM: atomic force microscopy; AsH_3_: arsine; C1: first electron sub-band; FP: Fabry-Perot; FTIR: Fourier-transform infrared; HH1: first heavy hole sub-band; HRXRD: high-resolution X-ray diffraction; IR: infrared; LH1: first electron sub-band; LO: longitudinal-optical; MBE: molecular beam epitaxy; MOCVD: metalorganic chemical vapor deposition; QWIP: quantum well IR photodetectors; RMS: root mean square; SL: superlattice; TMSb: trimethylantimony.

## Competing interests

The authors declare that they have no competing interests.

## Authors' contributions

LGL carried out the experimental analysis and drafted the manuscript. SML carried out the experimental design. SL carried out the growth and optimization of InAs/GaSb superlattice. TY and LJW participated in the experimental analysis. FQL and XLY participated in the experimental measurement. BX and ZGW participated in the experimental design. All authors read and approved the final manuscript.
